# Integrative Proteomics and *N*-Glycoproteomics Analyses of Rheumatoid Arthritis Synovium Reveal Immune-Associated Glycopeptides

**DOI:** 10.1016/j.mcpro.2023.100540

**Published:** 2023-04-04

**Authors:** Zhiqiang Xu, Yi Liu, Siyu He, Rui Sun, Chenxi Zhu, Shuangqing Li, Shan Hai, Yubin Luo, Yi Zhao, Lunzhi Dai

**Affiliations:** 1 National Clinical Research Center for Geriatrics and Department of General Practice, State Key Laboratory of Biotherapy, West China Hospital, Sichuan University, and Collaborative Innovation Center of Biotherapy, Chengdu, China; 2 Department of Rheumatology and Immunology, West China Hospital, Sichuan University, Chengdu, China

**Keywords:** rheumatoid arthritis, synovium, protein glycosylation, *N*-glycoproteomics, immune cell infiltration

## Abstract

Rheumatoid arthritis (RA) is a typical autoimmune disease characterized by synovial inflammation, synovial tissue hyperplasia, and destruction of bone and cartilage. Protein glycosylation plays key roles in the pathogenesis of RA but in-depth glycoproteomics analysis of synovial tissues is still lacking. Here, by using a strategy to quantify intact *N*-glycopeptides, we identified 1260 intact *N*-glycopeptides from 481 *N*-glycosites on 334 glycoproteins in RA synovium. Bioinformatics analysis revealed that the hyper-glycosylated proteins in RA were closely linked to immune responses. By using DNASTAR software, we identified 20 *N*-glycopeptides whose prototype peptides were highly immunogenic. We next calculated the enrichment scores of nine types of immune cells using specific gene sets from public single-cell transcriptomics data of RA and revealed that the *N*-glycosylation levels at some sites, such as IGSF10_N2147, MOXD2P_N404, and PTCH2_N812, were significantly correlated with the enrichment scores of certain immune cell types. Furthermore, we showed that aberrant *N*-glycosylation in the RA synovium was related to increased expression of glycosylation enzymes. Collectively, this work presents, for the first time, the *N*-glycoproteome of RA synovium and describes immune-associated glycosylation, providing novel insights into RA pathogenesis.

## Highlights

• An atlas of the proteome and *N*-glycoproteome of RA and OA synovium.
• Identification of 15 prototype peptides of *N*-glycopeptides with good immunogenicity.
• Identification of 5 *N*-glycopeptides correlated with certain immune cells infiltration.
• Aberrant *N*-glycosylation is related to elevated glycosylation enzymes in RA synovium.

## Graphical Abstract

**Figure:**
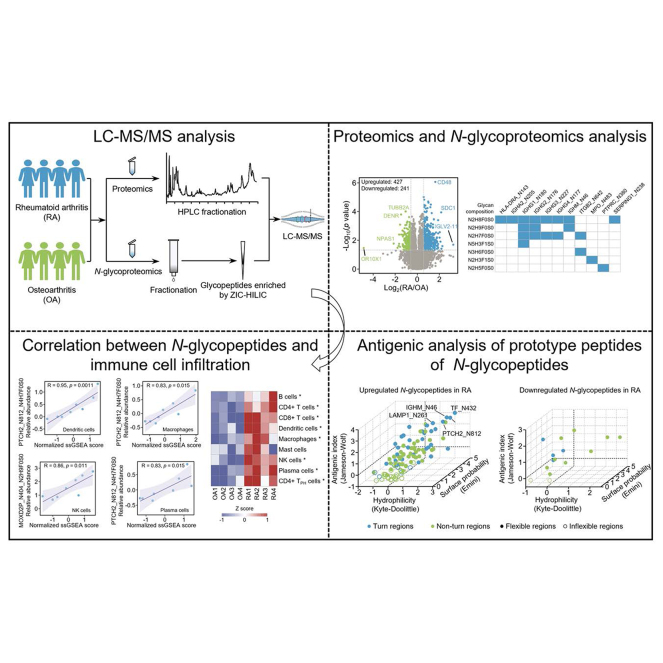

## In Brief

Protein glycosylation plays key roles in the pathogenesis of RA but in-depth glycoproteomics analysis of synovial tissues is still lacking. Here, we established the global *N*-glycoproteomics landscape of synovium from RA and OA patients by using a TMT-based strategy. Our results revealed that the hyper-glycosylated proteins in RA were closely linked to immune responses. We identified some N-glycopeptides correlated with the filtration of certain immune cells. This work identifies immune-associated *N*-glycoproteomics signatures of RA synovium.

Rheumatoid arthritis (RA) is a typical autoimmune disease (1). The etiology of RA is multifactorial. To date, a great number of susceptibility genes (*e.g.*, MHC-shared epitope, PADI and PTPN22), environmental factors (*e.g.*, periodontitis, oral microbiome, bronchial microbiome, gut microbiome, and smoking), and aberrant protein posttranslational modifications (*e.g.*, citrullination and carbamylation) have been identified as risk factors for RA (1, 2, 3, 4). However, the prevention and treatment of RA are still challenging due to the complex and elusive mechanisms. It is common for RA patients to have no improvement on medication or no continued remission after medication discontinuation (3, 5). In recent years, thanks to the rapid development of cutting-edge omics technologies, such as genomics (6), transcriptomics (7), proteomics (8), citrullinomics (9), carbamylomics (10), and single-cell transcriptomics (11), important progress has been made in the understanding of RA mechanisms.

Protein glycosylation is a widely distributed posttranslational modification involved in various intracellular and extracellular biological processes (BPs), and subtly regulates protein conformation, stability, activity, transport, and interaction with other molecules (12, 13). In innate and adaptive immunity, a great number of key proteins are glycosylated, such as Igs and most complement components involved in humoral immunity (14, 15). In the process of antigen presentation and activation of cytotoxic T cells, protein glycosylation can affect antigen processing by blocking the hydrolysis of protein antigens (16). Accumulating evidence suggests that antibody glycosylation is critical in autoimmunity but the links between the changes in autoimmunity-related glycosylation and disease pathology have not yet been fully elucidated (17). *N*-Glycoproteins are often present in the extracellular space and on the cell surface, which makes them advantageous as disease biomarkers and drug targets (18).

Autoimmune diseases usually have unique glycosylation characteristics (19). A close relationship between glycosylation and RA has been disclosed. Increasing evidence shows that many factors, such as age, sex, and disease severity, have impacts on glycosylation in RA (20, 21, 22). For example, dysregulated IgG galactosylation has been found to be associated with disease activity in a sex-dependent manner. However, the associations between the severity of RA and specific glycosylation sites remain poorly studied. A previous study showed that approximately 3 months before the onset of RA, sialylation and galactosylation of the fragment crystallizable (Fc) of anticitrullinated protein antibodies (ACPAs) are significantly reduced, a manifestation of increased systemic inflammation. In contrast, core fucose modifications increase in the first few years of RA onset (23). The *N*-linked glycans in the variable domain of ACPAs are also important to the development of RA (4, 24, 25, 26) and serve as potential RA biomarkers. Glycosylation of the IgG variable domain on the surface receptors of B cells affects its binding with autoantigens and alters autoreactive B-cell activation in RA (27). In addition, glycosylation of monomeric serum IgA2 also affects proinflammatory neutrophils and macrophages in patients with RA, leading to higher disease activity (28). Therefore, IgG glycosylation is used to assess disease activity and drug response in RA (17, 29).

The synovium is the main site of the inflammatory process in RA. Given the close relationship between glycosylation and inflammation, we speculate that synovial glycosylation may also have critical but uncharacterized functions. Over the past few years, cutting-edge techniques for characterizing intact glycopeptides have been developed (30, 31, 32, 33, 34, 35, 36, 37) and widely used in studying cancers (38, 39, 40), immune diseases (41, 42, 43), viruses (44, 45), Alzheimer’s disease (46, 47), cardiovascular disease (48, 49, 50), and male infertility (51). Despite tremendous progress, the synovial precision *N*-glycoproteome and its clinical significance in RA are completely unknown. In this study, for the first time, we performed *N*-glycoproteomics analysis of RA and osteoarthritis (OA) synovium and identified many immune-associated *N*-glycopetides, providing new insights into RA pathogenesis.

## Experimental Procedures

### Declaration of Helsinki Principles

RA and OA synovial tissues were collected with the approval of the Ethics Committee on Biomedical Research, West China Hospital of Sichuan University (Permission number: 2020(105)). Synovial proteomics and *N*-glycoproteomics analyses were conducted according to the Declaration of Helsinki ethical principles.

### Experimental Design and Statistical Rationale

Protein glycosylation is important for the occurrence and progression of RA. Previous studies have disclosed the glycosylation profiles of IgG in serum/synovial fluid, APCA autoantibodies, and synovial fibroblasts (52, 53, 54). However, in-depth profiling of the *N*-glycoproteome in RA synovial tissues has never been carried out to date. In this work, we performed tandem mass tag (TMT)-based proteomic and *N*-glycoproteomic analyses using four-knee synovial tissues from RA patients. Four-knee synovial tissues from OA patients were used as controls. The disease duration of all recruited RA and OA patients was more than 36 months. To determine the comparability of proteomics and *N*-glycoproteomics data between the OA and RA groups based on our sample size, we first set the α error to 0.05 using a two-sided test and calculated the effect size. Then, the statistical power (1-β) of each protein or *N*-glycopeptide was calculated using GPower software (version 3.1) (https://www.psychologie.hhu.de/arbeitsgruppen/allgemeine-psychologie-und-arbeitspsychologie/gpower). OA is a degenerative disease of local joints with a much lower inflammation level than RA and is usually used as a control in RA studies (55). The TMT-labeled samples were prefractionated before MS analysis to improve proteome coverage and reduce interference. The proteomics was analyzed in technical duplicates. Pearson correlation analysis was carried out to assess the technical reproducibility of the proteomics data. For proteins identified in duplicate, protein abundance was calculated using their mean abundance. For proteins identified in one of the two replicates, the protein abundance was calculated using the result from that replicate. The *N*-glycopeptides were enriched with ZIC-HILIC tips before MS analysis. Proteins and *N*-glycopeptides with a ratio of RA/OA >1.5 or <0.67 and *p* value <0.05 (Student’s *t* test) were defined as significantly changed proteins and *N*-glycopeptides in RA. Orthogonal partial least squares discriminant analysis (OPLS-DA) was applied to evaluate the difference between RA and OA. The fold change statistics of proteins and *N*-glycopeptides were performed in Excel. A volcano plot was used to show the significantly changed proteins and *N*-glycopeptides between RA and OA. Gene set enrichment analysis was used for pathway enrichment. Gene annotation, including BP, molecular function, and cellular component, and Kyoto Encyclopedia of Genes and Genomes pathway were performed using DAVID 2021. Estimation of STromal and Immune cells in MAlignant Tumor tissues using Expression data (ESTIMATE) and single sample gene set enrichment analysis methods were used to analyze immune cell infiltration and immune cell enrichment scores in synovial tissues. To reveal the immune-associated *N*-glycopeptides, antigenic analysis of prototype peptides of *N*-glycopeptides was first carried out using DNASTAR software (https://www.dnastar.com/), followed by correlation analysis between the levels of *N*-glycopeptides and enrichment scores of different immune cells. The enrichment scores of nine types of immune cells were calculated using gene sets from the single-cell transcriptome data of RA synovial tissues (11). The other R packages, including ggplot2, reshape2, dplyr, ggalluvial, and scatterplot3d, were used for data processing and presentation.

### Clinical Sample Collection

RA patients and knee OA patients were diagnosed by rheumatologists according to the 1987 American College of Rheumatology criteria for RA and the American College of Rheumatology criteria for knee OA, respectively. Key clinical indicators, such as rheumatoid factor, anticyclic citrullinated peptide antibody, C-reactive protein), erythrocyte sedimentation rate, and/or joint involvement, were measured and considered during the diagnosis of RA. All synovial tissues were obtained from the knee joints of diagnosed RA and OA patients who underwent surgery at West China Hospital of Sichuan University. The detailed clinical information of all RA and OA cases is presented in supplemental Table S1. To avoid blood interference and protein degradation, all synovial tissues were washed with cold PBS, blotted dry, and immediately frozen in liquid nitrogen within 30 min after surgery. Then, all synovial tissues were stored at −80 °C until use.

### Protein Extraction and Digestion

The synovial tissues were lysed in RIPA buffer (50 mM Tris (pH = 7.4), 150 mM NaCl, 0.5% sodium deoxycholate (w/v), 1% NP-40 (v/v)) containing protease, and phosphatase inhibitors on ice and homogenized twice by a Gentle-MACS dissociator (Miltenyi Biotec GmbH) under the procedure “Protein_01_01”. The crude lysates were sonicated at 3 s on and 10 s off for 5 min with 195 W of JY92-IIN (NingBoXinYi) in ice water and centrifuged (20,000 rcf, 4 °C, 30 min). The supernatant was collected and the protein concentration was measured by the Bradford assay (Bio-Rad). Then, 70 μg of protein from each sample was reduced with 10 mM Tris (2-carboxyethyl) phosphine (TCEP) at 56 °C for 1 h and alkylated with 17 mM iodoacetamide in the dark at room temperature for 30 min to block free cysteine residues. After alkylation, the proteins were precipitated with methanol/chloroform/water (CH_3_OH:CHCl_3_:H_2_O = 4:1:3). The precipitate was air-dried and subjected to enzymatic digestion with trypsin at a 1:50 (w/w, trypsin/protein) ratio in 100 mM triethylammonium bicarbonate buffer for approximately 14 h. The trypsin was deactivated and the digested peptides were used for later isobaric labeling and analysis.

### Isobaric Labeling

TMT reagents were dissolved in anhydrous MS-grade acetonitrile (ACN) after equilibration to room temperature. The tryptic peptides of the samples were labeled with 10-plex TMT reagents according to the manual (Thermo Fisher Scientific). The labeling was incubated for 1 h at room temperature and then quenched with 5% hydroxylamine. TMT-labeled peptides of RA and OA samples were equally mixed and dried with a SpeedVac. The mixed peptides were divided into two parts after reconstitution with 0.1% trifluoroacetic acid (TFA). Six percent of the combined sample was used for proteomics analysis and the rest was used for *N*-glycoproteomics analysis.

### Peptide Fractionation

The peptides used for proteomics analysis were desalted with a C18 SPE column (TECAN, CEREX 10 mg) and then fractionated by reversed-phase-HPLC (SHIMADZU LC-2030 plus) on a 25-cm reversed-phase C18 column (4 μM, 4.6 × 250 mm, Poroshell, Agilent) under basic pH. The separation was performed at a flow rate of 1 ml/min, the column temperature was kept at 40 °C, and UV detection was set at 214 nm. Gradient elution was carried out with a mixture of buffer A (98% H_2_O with 2% ACN, 10 mM ammonium formate, pH = 10) and buffer B (90% ACN with 10% H_2_O, 10 mM ammonium formate, pH = 10). A standard 120 min LC gradient run was used: 0 to 90 min, 3% to 35% buffer B; 90 to 105 min, 35% to 60% buffer B; 105 to 115 min, 60% to 100% buffer B; and 115 to 120 min, 100% to 3% buffer B. The peptide mixture was separated into 120 fractions and combined into 20 fractions. The combined fractions were dried by SpeedVac and desalted with C18 ZipTip before LC‒MS/MS analysis.

The peptides used for *N*-glycoproteomics analysis were fractionated by a C18 SPE column (Waters, Sep-Pak C18 100 mg) with a mixture of buffer A (98% H_2_O with 2% ACN, 10 mM ammonium formate, pH = 10) and buffer B (90% ACN with 10% H_2_O, 10 mM ammonium formate, pH = 10) under basic pH. For the SPE column fractionations, cartridges were preconditioned using 1 ml methanol and equilibrated with 1 ml buffer A twice. The peptide mixture was loaded onto an SPE column and this step was repeated 3 times. Gradient elution was carried out as follows: 2%, 4%, 6%, 8%, 10%, 12%, 14%, 16%, 18%, 20%, 22%, 25%, 30%, 50%, and 80% buffer B. Each gradient was eluted with 450 μl of buffer and the eluate was collected. The peptide mixture was divided into 15 fractions, which were further combined into five fractions. The combined fractions were dried by a SpeedVac and desalted with a C18 SPE column (TECAN, CEREX 10 mg) before *N*-glycopeptide enrichment.

### N-Glycopeptide Enrichment

Intact *N*-glycopeptides were enriched using home-made StageTips packed with ZIC-HILIC particles (Merck Millipore, particle size 5 μm, pore size 200 Å) as described previously (56). The desalted peptides were resuspended in loading buffer (80% ACN with 5% TFA). Before enrichment, StageTip was activated with H_2_O, 0.5 M NaCl, H_2_O and 80% ACN successively and equilibrated with 0.1% TFA, followed by loading buffer. The peptide mixture was loaded onto the ZIC-HILIC tip. After washing with the loading buffer, the *N*-glycopeptides were eluted with 100 μl 0.1% TFA three times, 50 μl 50 mM NH_4_HCO_3_ twice and 50 μl 50% ACN twice. The eluants for each fraction were combined and dried by SpeedVac. All the fractions were desalted with C18 ZipTip before LC‒MS/MS analysis.

### LC‒MS/MS Analysis

For proteomics analysis, the desalted peptides were resuspended in buffer A (2% ACN, 0.1% formic acid) and loaded onto a home-made trap column (2.5 cm length × 75 μm inner diameter, Spursil C18 5 μm particle size, DIKMA) coupled to a homemade capillary column (25 cm length × 75 μm inner diameter, Reprosil-Pur C18-AQ 1.9 μm particle size, Dr Maisch). LC‒MS/MS analysis was performed using an EASY-nanoLC 1200 nanoflow LC instrument coupled to a high-resolution mass spectrometer (Q Exactive HF-X, Thermo Fisher Scientific). Peptides were separated and eluted with a gradient of 13% to 100% HPLC buffer B (0.1% formic acid in 80% acetonitrile, v/v) in buffer A (0.1% formic acid in 98% water, v/v) at a flow rate of 330 nl/min. Data-dependent acquisition was performed in positive ion mode. Full MS was acquired in the Orbitrap mass analyzer over the range m/z 350 to 1600 with a resolution of 60,000 at m/z 200. The automatic gain control (AGC) value was set at 3e6 with a maximum injection time of 20 ms. The top 20 most intense parent ions were selected for MS/MS scans with a 0.6 m/z isolation window and fragmented with a normalized collision energy of 30%. The AGC value for MS/MS was set to a target value of 1e5, with a maximum injection time of 64 ms and a resolution of 30,000. Parent ions with a charge state of z = 1 or 8 or with unassigned charge states were excluded from fragmentation and the intensity threshold for selection was set to 3.1e5.

For *N*-glycoproteomics analysis, the enriched peptides were analyzed using an EASY-nanoLC 1000 nanoflow LC instrument coupled to a high-resolution mass spectrometer (Q Exactive Plus, Thermo Fisher Scientific). The peptides were separated with a gradient of 13% to 100% HPLC buffer B (0.1% formic acid in 80% ACN, v/v) in buffer A (0.1% formic acid in 98% water, v/v) at a flow rate of 330 nl/min. Data-dependent acquisition was performed in positive ion mode. Full MS was acquired in the Orbitrap mass analyzer over the range m/z 350 to 1600 with a resolution of 70,000 at m/z 200. The AGC value was set at 3e6 with a maximum injection time of 20 ms. The top 20 most intense parent ions were selected for MS/MS scans with a 0.6 m/z isolation window and fragmented with stepped normalized collision energies of 20/27/31%. The AGC value for MS/MS was set to a target value of 1e5, with a maximum injection time of 100 ms and a resolution of 35,000. Parent ions with a charge state of z = 1 or 6 to 8 or with unassigned charge states were excluded from fragmentation, and the intensity threshold for selection was set to 2e5.

### MS Data Processing

For proteomics data, all the raw files were searched against the Swiss-Prot human protein sequence database (updated on 01/2017; 20,413 protein sequences) by using MaxQuant (version 1.6) (https://www.maxquant.org/maxquant/). The precursor ion mass errors of all the identified peptides were within 10 ppm and the fragment ion mass tolerance was set at 0.02 Da. Cysteine alkylation by iodoacetamide was specified as a static modification and methionine oxidation and protein N-terminal acetylation were specified as variable modifications. The minimum peptide length was set at six amino acids. The max missed trypsin cleavages were set at two and peptides were not nested within another longer peptide. Proteins with a false discovery rate (FDR) <1% at both the protein and peptide levels were kept. After removing the contaminant and reverse proteins, the mean protein abundance in duplicate was calculated. Then, the total protein intensities of each sample were normalized to the same level. To assess the technical reproducibility of the proteomics data, Pearson correlation analysis was carried out. To assess the homogeneity of the OA and RA samples, OPLS-DA of the proteomic data was performed.

For *N*-glycoproteomics, the intact *N*-glycopeptides were searched based on a theoretical *N*-glycan library, which was built according to currently known biosynthesis rules and retrosynthetic strategies and contains 108,921 polysaccharide sequence structures, by GPSeekerPro (57). The protein sequence database used for *N*-glycoproteomics was the same as that used in proteomics. To search the matched precursors and fragment ions, the minimum peptide length was set at six amino acids, the isotopic peak abundance cutoff was set at 20%, the isotopic peak m/z deviation was set at 20 ppm, and the isotope peak abundance deviation was set at 50%. To search the intact *N*-glycopeptide spectra, the minimum percentage of matched fragment ions of the peptide backbone was set at 10% and the minimum matched fragment ions of the *N*-glycan moiety was set at 1. The FDR of intact *N*-glycopeptides was assessed with the widely adopted target-decoy search strategy and scoring system (P score), and a threshold P score was chosen to give a spectrum-level FDR of no more than 1%, which was computed with 2∗nf∗100%/nr (nf is the number of *N*-glycopeptide spectrum matches (GPSMs) below the threshold P score from the forward search, and nr is the number of GPSMs below the threshold P score from the decoy search). The linkage structures of *N*-glycans in the GPSeekerPro software are built with the currently known biosynthetic rules (58, 59) and the retro-synthetic strategy (60). For GPSMs with a control spectrum-level FDR ≤1%, a unique intact *N*-glycopeptide is distinguished by combining the peptide sequence, *N*-glycan linkage, and *N*-glycosite information. The *N*-linked glycan structure-diagnostic ions score (GF score) was used to assess the diagnostic ions of the *N*-linked glycan structure. However, some *N*-glycopeptides with the same amino acid sequence and composition may not be separated by LC, resulting in a mixed spectrum. Therefore, only suggested glycan peptides were provided in this work to avoid any inaccurate annotation, although some diagnostic ions for *N*-linked glycan structure could still be well determined. The intact *N*-glycopeptide was determined using the peptide with *N*-glycan composition, which was further used for the presentation of data. Before data analysis, the intensity of the *N*-glycopeptide was normalized to the intensity of the corresponding protein in each sample and the resulting datasheet was used for further data analyses. To assess the homogeneity of OA and RA samples, OPLS-DA of the *N*-glycoproteomic data was performed.

### Antigenicity Analysis

To perform peptide antigenicity analysis, we first extracted the amino acid sequence around the *N*-glycosite (centered on Asn, 15 amino acids on the left and right). By using DNASTAR software, the flexibility (Karplus–Schulz), β-turns (Garnier–Robson), hydrophilicity (Kyte–Doolittle), protein surface accessibility (Emini), and peptide antigen index (Jameson–Wolf) of *N*-glycopeptides were calculated. We screened antigenic peptides with flexible regions, β-turns, strong hydrophilicity (Kyte–Doolittle score ≥ 1), a more likely protein surface region (Emini score ≥ 1), and a high antigenic index (Jameson–Wolf ≥ 1).

### Immunofluorescence Staining

All synovial specimens diagnosed as OA and RA by pathologists were made into donor paraffin blocks. Immunofluorescence staining was performed as previously described (61). The total T cell population was determined using a CD3 antibody, whereas the CD4^+^ and CD8^+^ T cell populations were assessed using CD4 and CD8 antibodies, respectively. The B cell, NK cell, macrophage, and dendritic cell populations were determined using CD19, CD56, CD68, and CD83 antibodies, respectively. The quantitative results of immunofluorescence staining were obtained upon microscopy image by using the available tools of Fiji/ImageJ software (https://imagej.net/imagej-wiki-static/Fiji). The total number of cells was determined based on 4',6-diamidino-2-phenylindole nuclear staining, and CD3-, CD4-, CD8-, CD19-, CD56-, CD68-, and CD83-positive cells were counted based on red and green fluorescence. The immunofluorescence results were used to assess the extent and features of infiltrating immune cells.

## Results

### Proteomics and *N*-Glycoproteomics Profiling in RA and OA Tissues

**Fig. 1:**
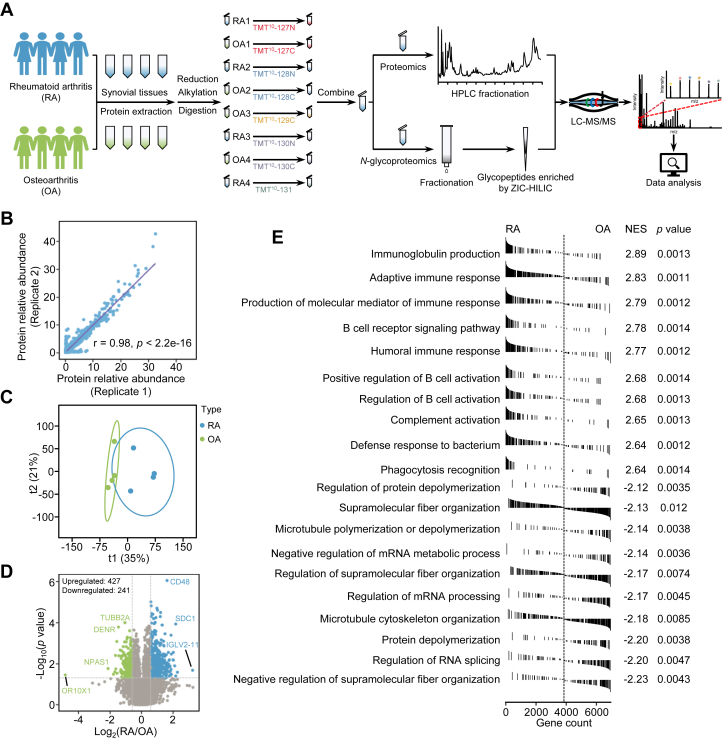
**Quantitative proteomics analysis of OA and RA synovial tissues.***A*, the workflow of quantitative proteomics and *N*-glycoproteomics analyses of human synovium. *B*, Pearson correlation analysis of the proteomics technical duplicates. *C*, OPLS-DA score plots of proteomics data of RA and OA. *D*, volcano plot showing 427 upregulated and 241 downregulated proteins in RA (median ratio (RA/OA) >1.5 or <0.67, and Student’s *t* test, *p* < 0.05). *E*, GSEA preranked analysis of identified proteins in RA and OA. NES represents the normalized enrichment score. GSEA, gene set enrichment analysis; OA, osteoarthritis; OPLS-DA, orthogonal partial least squares discriminant analysis; RA, rheumatoid arthritis.

Fresh knee joint synovial tissues of four patients with OA and four patients with RA were used. A TMT-based quantitative proteomics and *N*-glycoproteomics strategy was applied to quantify the differences in proteins and *N*-glycosylation between RA and OA (Fig. 1*A*). As a result, a total of 7227 proteins with FDR less than 1% in duplicates were quantified (supplemental Table S2 and Fig. 1*B*). OPLS-DA based on the proteomics data showed that the RA and OA samples could be well separated (Fig. 1*C*). Comparative analysis identified 427 and 241 proteins upregulated and downregulated in RA, respectively (fold change >1.5, and Student’s *t* test, *p* < 0.05) (Fig. 1*D* and supplemental Table S3). Gene set enrichment analysis of upregulated proteins in RA revealed that these proteins were linked to immunity (Fig. 1*E*), confirming the overactivated immune system in RA synovium.

**Fig. 2:**
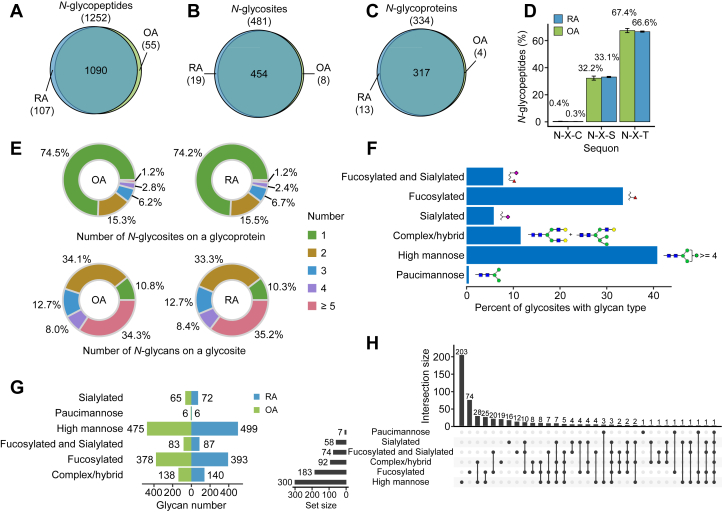
**Characteristics of intact *N*-glycopeptides in RA and OA synovium.***A*–*C*, Venn diagram showing identified *N*-glycopeptides (*A*), *N*-glycosites (*B*), *N*-glycoproteins, and (*C*) in RA and OA samples. *D*, distribution of all identified *N*-glycopeptides with N-X-T/S/C tripeptide sequons is shown as the means ± SDs (n = 4 cases per OA or RA group). *E*, pie charts depict the percentage of *N*-glycoproteins with the identified number of *N*-glycosites per protein (*upper*) and the percentage of *N*-glycosites with the identified number of *N*-glycans per glycosite in OA and RA samples (*below*). *F*, percentage of the six types of glycans in all *N*-glycosites. *G*, number of *N*-glycosites with different glycan types identified in the OA and RA groups. *H*, UpSet plot represents the frequency of glycan pairs existing at the same *N*-glycosite. OA, osteoarthritis; OPLS-DA, orthogonal partial least squares discriminant analysis; RA, rheumatoid arthritis.

In parallel, by using GPSeekerPro software (36), we identified 1260 confident intact *N*-glycopeptides derived from 481 distinct *N*-glycosites on 334 *N*-glycosylated proteins and 2595 suggested *N*-glycan structures were provided (Fig. 2, *A*–*C* and supplemental Table S4). All the *N*-glycopeptides satisfied the canonical *N*-glycosylation motif (seqon) N-X-S|T|C (where X represents an amino acid other than proline). Statistical analysis showed a more frequent N-X-T motif (∼67%) than the N-X-S motif (∼33%), while the N-T-C motif accounted for approximately 0.3% of the total (Fig. 2*D*). Distribution analysis showed that more than half of the *N*-glycosylated proteins (∼74%) had only one *N*-glycosite, approximately 15% of the *N*-glycoproteins had two *N*-glycosites and approximately 10% of *N*-glycoproteins had more than two *N*-glycosites (Fig. 2*E*, upper). Notably, approximately 33% of *N*-glycosites had two glycans and approximately 34% of the *N*-glycosites had more than four glycans (Fig. 2*E*, below). Most likely due to the use of the TMT-based strategy by which all the RA and OA samples were analyzed in one batch, the identified *N*-glycosites on the same protein and the identified glycans on the same *N*-glycosite were almost identical between the RA and OA groups but the abundance of these *N*-glycopeptides was different between the two groups.

To explore the microheterogeneity of *N*-glycosites in RA and OA synovium, we next classified the suggested glycan structures into six groups according to the literature (47), including paucimannose, high mannose, complex/hybrid, sialylated, fucosylated, and fucosylated and sialylated glycans (Fig. 2*F*), and found that more than 68% of *N*-glycosites were modified by high mannose (32%) and fucosylated (36%) glycans (Fig. 2, *F* and *G*). The frequency of the simultaneous occurrence of the six types of glycans on the same *N*-glycosite was counted. Most *N*-glycosites had more than one type of glycan, with high mannose and complex type/hybrid glycans co-occurring on the same *N*-glycosites with the highest frequency (Fig. 2*H*). High mannose and complex/hybrid glycans are ubiquitous glycan types in humans and play key roles in cell migration, cell signaling, and antibody recognition (62, 63, 64). Taken together, we profiled the proteome and *N*-glycoproteome in RA and OA synovium and identified the most frequent glycans on the proteins of RA synovium.

### Differences in the *N*-Glycoproteome Between RA and OA

**Fig. 3:**
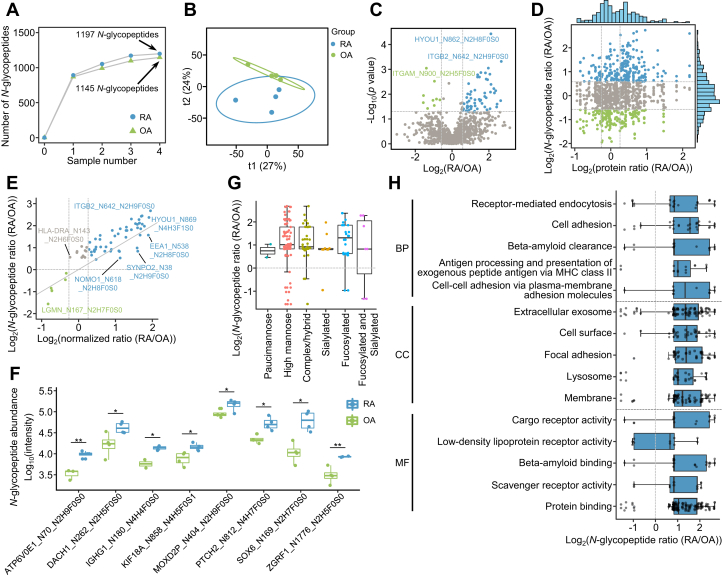
**Differences in the *N*-glycoproteome of RA and OA synovium.***A*, cumulative number of *N*-glycopeptides identified in OA (n = 4) and RA (n = 4) samples. *B*, OPLS-DA score plots of *N*-glycoproteomics data of OA and RA synovium. *C*, volcano plot showing 67 upregulated and nine downregulated *N*-glycopeptides in RA (median ratio (RA/OA) >1.5 or <0.67 and Student’s *t* test, *p* < 0.05). *D*, scatter plot depicting the differences in *N*-glycopeptides and their corresponding proteins between OA and RA. The *blue* and *green dots* represent the median ratio (RA/OA) of each *N*-glycopeptide greater than 1.5 and less than 0.67, respectively. The *dashed lines* represent the median fold change of proteins at 1.2 and the median fold change of *N*-glycopeptides at 1.5. *E*, scatter plot shows the correlation of *N*-glycopeptides with fold changes greater than 1.5 in (*C*) before and after normalization to glycoproteins. The *blue* and *green dots* represent the normalized ratio (RA/OA) of each *N*-glycopeptide greater than 1.2 and less than 0.83, respectively. The *gray solid line* represents the line with slope 1. *F*, box plot shows the differences in *N*-glycopeptides in (*C*) without proteins identified in proteomics (Student’s *t* test, ∗*p* < 0.05; ∗∗*p* < 0.01). *G*, comparison of the distribution of *N*-glycans on the significantly changed *N*-glycopeptides. *H*, comparison of *N*-glycoproteins related to different clusters based on cell compartment (CC), biological process (BP), and molecular function (MF). OA, osteoarthritis; RA, rheumatoid arthritis.

Comparative analysis identified 76 intact *N*-glycopeptides with significant abundance differences between RA and OA (fold change > 1.5 and Student’s *t* test, *p* < 0.05) (Fig. 3, *A*–*C*). Of these, 67 *N*-glycopeptides on 43 glycoproteins showed higher levels in RA, and 9 *N*-glycopeptides on nine glycoproteins had lower levels in RA (Fig. 3*C* and supplemental Table S5). Notably, alterations in most *N*-glycopeptides were independent of the corresponding protein differences (Fig. 3, *D* and *E*). In addition, we also identified some differentially intact *N*-glycopeptides of low-abundance proteins that were not identified by proteomics (Fig. 3*F*). Statistical analysis showed that a majority of *N*-glycosites with altered glycosylation in RA were modified by high mannose, complex type/hybrid, and fucosylated glycans (Fig. 3*G*). Localization analysis showed that most *N*-glycoproteins with increased glycosylation in RA were localized on the membrane, extracellular space, and exosomes, and these proteins were mainly involved in receptor-mediated endocytosis, antigen processing and presentation, and the immune response (Fig. 3*H*), suggesting the critical roles of protein glycosylation in the immunopathological processes of RA.

**Fig. 4:**
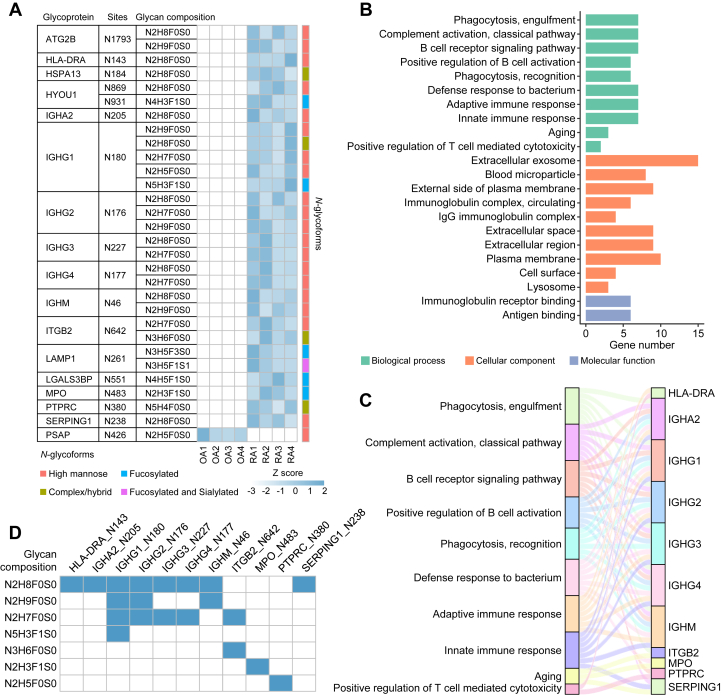
**RA- and OA-specific intact *N*-glycopeptides in synovium.***A*, heatmap showing the 29 RA- and 1 OA-specific *N*-glycopeptides with glycan compositions in synovium. The value for each *N*-glycopeptide is the adjusted intensity with a row-scaled Z score. *B*, GO analysis of the 16 glycoproteins derived from 29 RA-specific *N*-glycopeptides in terms of cellular component (CC), biological process (BP), and molecular function (MF). The top ten biological processes and cellular components are shown. *C*, Sankey diagram showing the 11 glycoproteins in the top ten biological processes of (*B*) and their interlinkages. *D*, heatmap representing the appearance of different glycan compositions at *N*-glycosites of the 11 glycoproteins in (*C*). OA, osteoarthritis; RA, rheumatoid arthritis.

Moreover, we also summarized *N*-glycopeptides that were specifically identified in RA or OA. RA-specific *N*-glycopeptides represented the peptides detected in no less than 75% of RA samples but not in any OA sample, and OA-specific *N*-glycopeptides represented the peptides detected in no less than 75% of OA samples but not in any RA sample. In total, 60 RA-specific and 4 OA-specific *N*-glycopeptides were obtained (supplemental Table S6). Of them, 29 RA-specific *N*-glycopeptides were identified in all RA samples but not in any OA sample, while only 1 OA-specific glycopeptide was identified in all OA samples but not in any RA sample (Fig. 4*A* and supplemental Table S6). Notably, a number of *N*-glycoproteins, such as the constant region of Ig heavy chains (IGHG1, IGHG2, IGHG3, IGHG4, and IGHA2), were modified by different glycans on the same *N*-glycosite and the glycan compositions were provided (Fig. 4*A*). Glycosylated HYOU1, which has been identified as a potential antitumor vaccine (65), was also detected in RA. ITGB2, which is associated with inflammatory responses and the adhesion and metastasis of leukocytes, is also modified by many types of glycans at N642 (66, 67). Enrichment analysis of 16 *N*-glycoproteins derived from the 29 RA-specific *N*-glycopeptides revealed that most glycoproteins were localized at the extracellular exosome, extracellular space, and plasma membrane and were involved in phagocytosis, complement activation, and adaptive and innate immune responses (Fig. 4, *A* and *B* and supplemental Table S6). To understand the contribution of RA-specific *N*-glycoproteins to different BPs, a Sankey diagram was used to show the 11 *N*-glycoproteins in the top ten pathways and their interlinkages (Fig. 4, *B* and *C*). By counting the glycans of the suggested structures on the 11 *N*-glycoproteins (Fig. 4*D*), we found that high mannose was the most frequent glycan on RA-specific glycoproteins. Collectively, these results indicate higher *N*-glycosylation in RA than in OA.

### Antigenicity Analysis of the Prototype Peptides of *N*-Glycopeptides

**Fig. 5:**
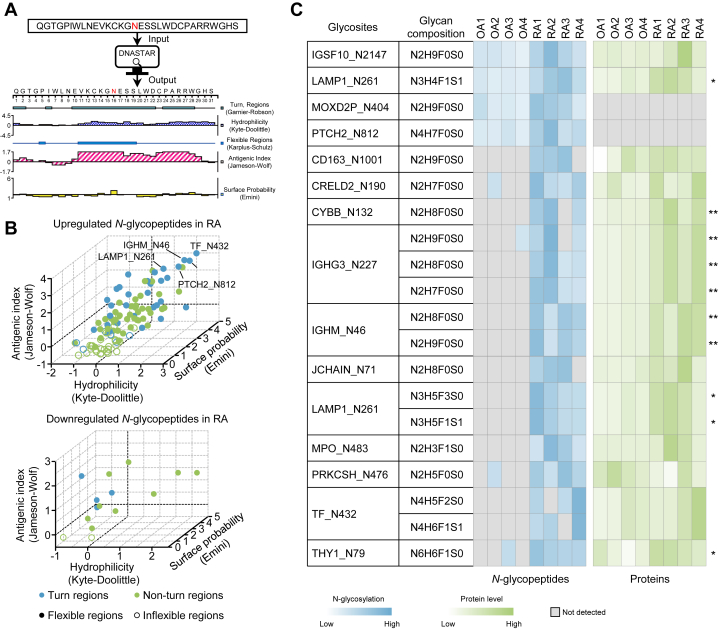
**Antigenic analysis of *N*-glycopeptides.***A*, a workflow of antigenicity prediction of 146 prototype peptides derived from differentially expressed *N*-glycopeptides between OA and RA by using DNASTAR software. *B*, 3D scatter plot showing the hydrophilicity, flexible regions, β-turns, surface probability, and antigenic index of upregulated and downregulated *N*-glycopeptides in the RA and OA groups. *C*, heatmap showing the abundances of 20 *N*-glycopeptides whose prototype peptides were predicted to have good antigenicity. The protein changes are shown on the *right* of the figure (Student’s *t* test, ∗*p* < 0.05; ∗∗*p* < 0.01). OA, osteoarthritis; RA, rheumatoid arthritis.

Previous studies have shown that glycosylation may affect antigen processing and presentation (16), which further impacts the onset and progression of autoimmune diseases. To reveal whether *N*-glycosylation has the potential to affect the antigenicity of amino acid sequences surrounding the modified sites, antigenicity analysis of the 146 prototype peptides derived from differentially expressed *N*-glycopeptides between OA and RA was performed using DNASTAR software (Fig. 5*A* and supplemental Table S7). The flexibility, β-turns, hydrophilicity, protein surface accessibility, and peptide antigen index of *N*-glycopeptides were systematically evaluated (Fig. 5*B*). It is well recognized that β-turns and flexible regions of peptides are helpful to improve their antigenicity. Moreover, the more hydrophilic the peptide is (Kyte-Doolittle score ≥ 1), the more likely it is to be on the surface of the protein (Emini score ≥ 1) and the higher the antigenic index of the peptide (Jameson-Wolf ≥ 1). Based on the above criteria, we obtained 20 *N*-glycopeptides whose prototype peptides had an antigenic index greater than 1 (Fig. 5*B* and supplemental Table S7). Among the 20 intact *N*-glycopeptides, the differences in *N*-glycosylation were more variable than the protein differences, with the most *N*-glycopeptides only detected in RA synovium (Fig. 5*C*). However, how *N*-glycosylation affects the function of these antigenic peptides and participates in the development of RA remains to be investigated.

### Association Between *N*-Glycosylation and Immune Cell Infiltration

**Fig. 6:**
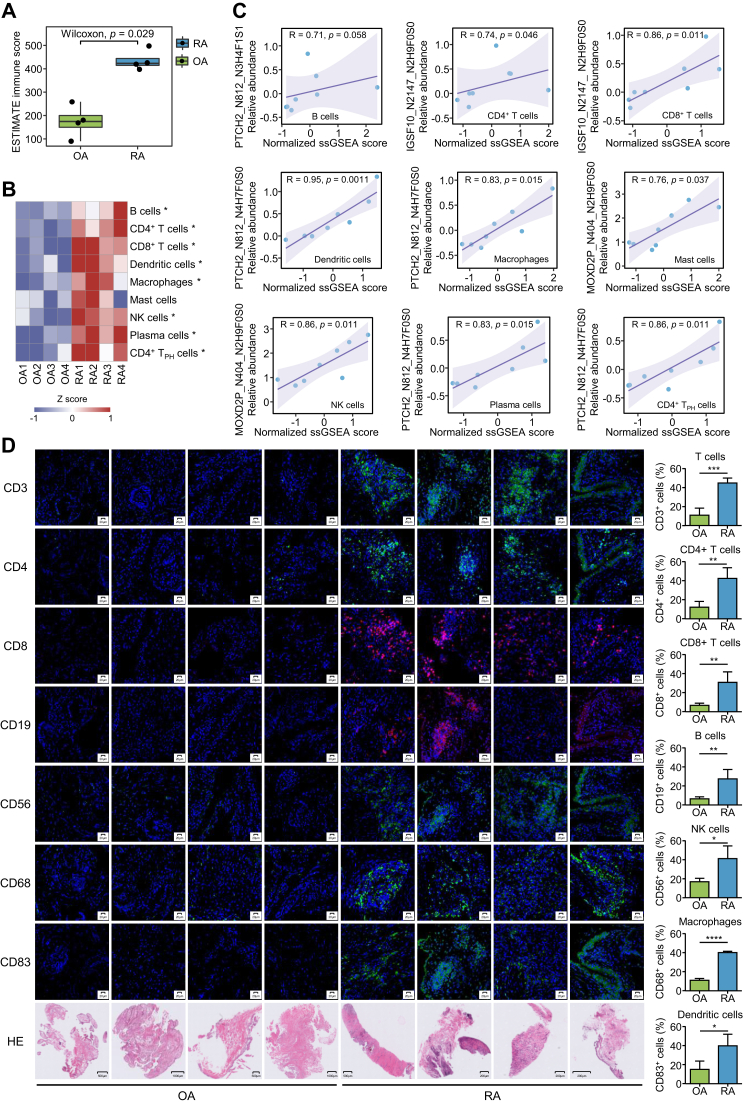
**Integrative multiomics analyses to identify immune-associated *N*-glycopeptides in RA.***A*, comparison of estimated immune scores between the RA and OA groups using the ESTIMATE algorithm based on the proteomics data. The box represents the median (*thick line*) and the quartiles (*line*). *B*, heatmap for row-scaled enrichment scores of nine immune cell types in synovial tissues. The enrichment scores were calculated by ssGSEA using specific gene sets for these immune cell types. *Rows* represent infiltrating immune cells and columns represent samples (Wilcoxon rank-sum test, ∗*p* < 0.05). *C*, representative differentially expressed *N*-glycopeptides show a significant linear correlation with the infiltration of immune cells. R represents the regression coefficient and the *p* value represents the significance of the linear correlation. *D*, H&E staining of synovial tissue sections and immunofluorescence staining of CD3, CD4, CD8, CD19, CD56, CD68, CD83, and the percentage of CD3, CD4, CD8, CD19, CD56, and CD68, CD83-positive cells in synovial tissues from OA (n = 4) and RA (n = 4) patients. The statistics of immune-positive cells were calculated using Fiji/ImageJ. Each value represents the average obtained from four independent experiments. Data are presented as the mean ± SD. (Student’s *t* test, ∗*p* < 0.05; ∗∗*p* < 0.01; ∗∗∗*p* < 0.001; ∗∗∗∗*p* < 0.0001). H&E staining of synovial tissue sections was used to observe biopsy pathological morphology. ESTIMATE, Estimation of STromal and Immune cells in MAlignant Tumor tissues using Expression data; OA, osteoarthritis; RA, rheumatoid arthritis; ssGSEA, single sample gene set enrichment analysis.

As an autoimmune disease, RA has a large number of immune cells infiltrating synovial tissues (11, 68). By using the ESTIMATE algorithm (69), the immune scores in RA and OA synovium were calculated based on the proteomics data (Fig. 6*A*). As expected, RA synovial tissues had significantly higher immune scores than OA synovial tissues. In addition, we used the widely applied single sample gene set enrichment analysis method to analyze the gene set enrichment scores of nine immune cell types (70), including B cells, CD4^+^ T cells, CD8^+^ T cells, dendritic cells, macrophages, mast cells, NK cells, plasma cells, and CD4^+^ T_PH_ cells, in the synovial tissue microenvironment of RA and OA (71) by using the representative gene sets derived from single-cell transcriptome data of RA synovium (11) (supplemental Table S8). We found that all these immune cell types showed higher enrichment scores in RA (Fig. 6*B*). Spearman correlation analysis of the levels of 20 *N*-glycopeptides listed in supplemental Table S7 with the enrichment scores of the nine immune cell types was carried out. We identified 5 *N*-glycopeptides that were significantly correlated with the enrichment scores of certain immune cell types (supplemental Table S9 and Fig. 6*C*), suggesting that differences in *N*-glycosylation may be intrinsically linked to the immune response in the synovium. To further verify the immune cell infiltration difference between OA and RA samples, we collected new synovial samples from diagnosed RA and OA cases to evaluate the infiltration of immune cells in OA and RA synovium. By performing immunostaining of T cells with the CD3 marker (72), B cells with the CD19 marker (73), NK cells with the CD56 marker (74), macrophages with the CD68 marker (75), and dendritic cells with the CD83 marker (76) in the OA and RA samples, we obtained consistent results that were calculated using the proteomics data and the publicly available immune cell gene sets (Fig. 6*D*).

### The Regulation of *N*-Glycosylation in the Synovium of RA Patients

**Fig. 7:**
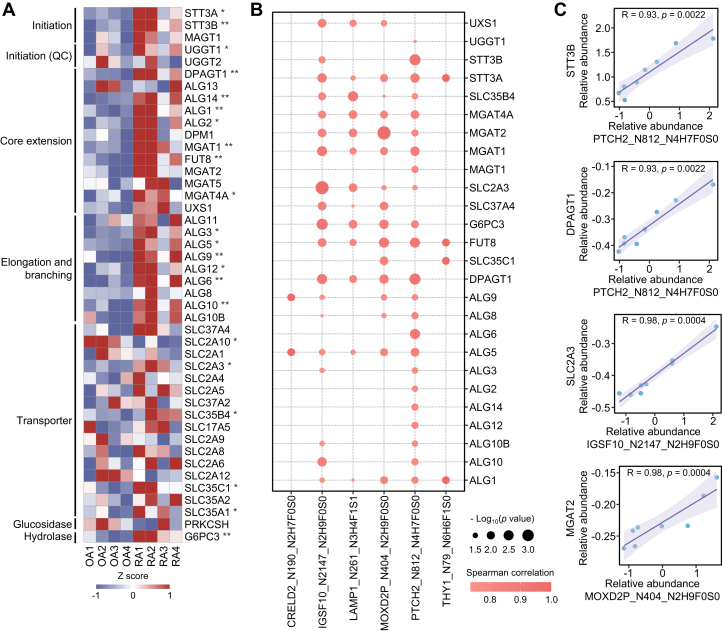
**Correlation analysis of *N*-glycopeptides and glycosylation enzymes.***A*, heatmap showing the expression of glycosylation-associated transferase, transporter, glucosidase, and hydrolase in RA and OA synovial tissues based on the proteomics data (Student’s *t* test, ∗*p* < 0.05; ∗∗*p* < 0.01). *B*, bubble chart shows differentially expressed *N*-glycopeptides and glycosylation enzymes with significant linear correlation (Spearman's rank correlation coefficient, *p* < 0.05). *C*, representative differentially expressed *N*-glycopeptides linearly correlated with glycosylation enzymes. R represents the regression coefficient and the *p* value represents the significance of the linear correlation. OA, osteoarthritis; RA, rheumatoid arthritis.

Protein *N*-glycosylation is the transfer of preassembled oligosaccharides from a dolichol-linked oligosaccharide donor to proteins with an N-X-S|T|C motif (X≠P) by OST located on the endoplasmic reticulum (77, 78, 79). In the glycosylation process, a series of enzymes are involved in glycan initiation, core extension, sugar chain elongation, and branching. Comparative analysis revealed that most enzymes associated with *N*-glycan initiation, core extension, elongation, and branching showed higher expression in RA than in OA, while the levels of most oligosaccharide and monosaccharide transporters showed no obvious difference between RA and OA (Fig. 7*A*). By performing correlation analysis of the levels of 20 *N*-glycopeptides with the expression of 44 glycosylation enzymes, we found that the *N*-glycosylation at CRELD2_N190, IGSF10_N2147, LAMP1_N261, MOXD2P_N404, PTCH2_N812, and THY1_N79 was significantly correlated with a series of glycosylation enzymes (Spearman's rank correlation coefficient, *p* < 0.05) (Fig. 7, *B* and *C*). The above results indicate that the increased levels of *N*-glycosylation in RA synovium may be caused by an aberrant increase in glycosylation enzymes.

## Discussion

In summary, for the first time, we presented an *N*-glycoproteome in synovial tissues from RA and OA patients and identified 1260 intact *N*-glycopeptides derived from 481 *N*-glycosites on 334 *N*-glycosylated proteins with 2595 suggested glycan structures in synovial tissues. Of these, 76 intact *N*-glycopeptides showed significant expression differences between RA and OA, with 60 *N*-glycopeptides specifically identified in RA. By performing antigenicity analysis, we identified 20 *N*-glycopeptides with prototype peptides showing good immunogenicity. Of the 20 *N*-glycopeptides, the levels of 5 *N*-glycopeptides were significantly correlated with the enrichment scores of certain immune cell types in the synovium, indicating that *N*-glycosylation at these sites may be involved in synovial immunity. Furthermore, we found that increased glycosylation enzymes in RA were associated with aberrant glycosylation, suggesting glycosylation enzymes as potential targets for RA treatment.

In recent decades, increasing evidence has shown that changes in the glycome and glycoproteome in body fluids and tissues are closely related to the occurrence and development of many autoimmune diseases. For example, glycoproteomics profiling of total serum IgG shows that OA and RA patients, especially RA patients, have reduced total serum IgG galactosylation levels compared with healthy individuals (52). A similar result has been observed in autoantibodies isolated from synovial fluid of RA (53). Interestingly, antibodies from different places have distinct glycosylation patterns. The level of *N*-glycosylation on the Fab domain of IgG from synovial fluid is significantly higher than that from plasma IgG (53). The Fc domain of ACPAs also exhibits different glycan profiles between synovial fluid and serum (22). Furthermore, glycomic analysis of synovial fibroblasts isolated from RA and OA patients revealed a reduction in sialylation on the cell surface of synovial fibroblasts in RA, which influenced the interaction between synovial fibroblasts and galectin-3 and led to the cytokine-induced proinflammatory phenotype (54). In other autoimmune diseases, such as systemic lupus erythematosus, inflammatory bowel disease, anti-neutrophil cytoplasmic antibody-associated vasculitis, fetal and neonatal alloimmune thrombocytopenia, and autoimmune liver disease, different types of IgG glycosylation show similar trends between these diseases. However, the levels of different glycosylation types on autoantibodies are between them (17).

Both RA and OA produce inflammation and lead to joint damage. OA is usually caused by physiological problems in the joints, while RA is an autoimmune disease with much higher levels of inflammation in synovial tissues. Our proteomics analysis showed that the upregulated proteins in RA synovium are associated with the immune response and complement activation (80, 81, 82), indicating that RA synovium is in a state of immune hyperactivation, which was further verified by immunostaining of immune cells in RA and OA knee synovial tissues (Fig. 6*D*). Subsequent analyses focused on the immune-related comparisons, which may be due to the difference in OA and RA N-glycosylation (Figs. 5 and 6), suggesting that the use of OA as a control for RA investigation in this work is reasonable.

Quantitative *N*-glycoproteomics revealed 76 *N*-glycosites with significant abundance differences in synovium between RA and OA. In addition, 29 *N*-glycopeptides were specifically detected in all RA patients but not in any OA patient, indicating that *N*-glycosylation at these sites is a potential biomarker for RA, which requires verification in large cohorts. By analyzing the antigenicity of 146 prototype peptides, 15 prototype peptides corresponding to 20 *N*-glycopeptides with high antigenicity were obtained. A previous study showed that glycosylation on antigens can be recognized by T cells and alterations in epitope glycosylation are involved in autoimmune diseases (16). There are two possible explanations. On the one hand, glycosylation on the surface of proteins may induce specific immune responses. For example, T cells from RA patients predominantly recognize the glycosylated form of the collagen type II epitope instead of its unmodified form (83). On the other hand, changes in glycosylation may alter the conformational equilibrium of epitopes, thus altering their immunogenicity (84). In this study, by analyzing immune infiltration in RA synovial tissues using the ESTIMATE algorithm, we found that the immune scores of RA synovial tissues were significantly higher than those of OA synovial tissues, consistent with the immunostaining results of RA and OA synovial tissues (Fig. 6*D*). Moreover, the levels of *N*-glycosylation at multiple *N*-glycosites, such as PTCH2_N812, MOXD2P_N404, and IGSF10_N2147, are significantly correlated with the enrichment scores of certain types of immune cells, suggesting that different *N*-glycopeptides may affect immune cells *via* different mechanisms.

Notably, we detected distinct glycan structures for IgG. On the one hand, the biantennary and bisected core-fucosylated glycan for IgG reported in some other studies (43, 52, 85, 86) was not identified in our study. On the other hand, the novel antennary fucose and triantennary glycan structure for IgG1 shows a significant difference between RA and OA samples, which has never been identified by others. We speculate that the different types of samples used may be one important reason for the different results. In the previous glycosylation profiling of IgG, serum samples were used, whereas our glycosylation profiling was performed using synovial tissues. A previous study showed that the Fc domain of ACPAs exhibits distinct glycan profiles between synovial fluid and serum (22), indicating that the source of the samples has a significant impact on the glycosylation profiles.

Of course, there are still some limitations in this study. First, the theoretical sequence and linkage structures of *N*-glycans are built with the currently known biosynthetic rules (58, 59) and the retro-synthetic strategy (60). Only a portion of the glycan structures were confirmed by structural diagnostic fragment ions. Moreover, combinatorial adjacent *N*- and *O*-glycosylation may simultaneously exist on the same tryptic intact *N*-glycopeptide, which cannot be well characterized in this work. Second, the proteomic and *N*-glycoproteomic signatures of synovial tissues reflect the difference between OA and RA. More efforts need be made to profile the RA-related *N*-glycosylome in larger RA cohorts with healthy or disease-free control samples. Third, the functions of immune-related *N*-glycopeptides remain to be disclosed. Collectively, this work establishes a link between synovial glycosylation and RA, providing new insights into RA pathogenesis.

## Data Availability

The mass spectrometry proteomics and *N*-glycoproteomic data have been deposited to the ProteomeXchange Consortium (http://proteomecentral.proteomexchange.org) *via* the iProX partner repository (87) with the dataset identifier PXD037581.

## Supplemental data

This article contains supplemental data.

## Conflict of interest

The authors declare no competing interests.
