# Supplementary material for: Integrative Proteomics and N-Glycoproteomics Analyses of Rheumatoid Arthritis Synovium Reveal Immune-Associated Glycopeptides
Source: Mol Cell Proteomics. 2023 Apr 4;22(5):100540. doi: 10.1016/j.mcpro.2023.100540 (PMC10176071; doi:10.1016/j.mcpro.2023.100540)
Supplement: Supplemental Information [file mmc1.docx]

*Supplemental information*

**Integrative proteomics and *N*-glycoproteomics analyses of rheumatoid arthritis synovium reveal immune-associated glycopeptides**

Zhiqiang Xu,^1,†^ Yi Liu,^2,†^ Siyu He,^1,†^ Rui Sun,^2,†^ Chenxi Zhu,^2^ Shuangqing Li,^1^ Shan Hai,^1^ Yubin Luo,^2^ Yi Zhao,^2,^* Lunzhi Dai^1,^*

^1^National Clinical Research Center for Geriatrics and Department of General Practice, State Key Laboratory of Biotherapy, West China Hospital, Sichuan University, and Collaborative Innovation Center of Biotherapy, Chengdu, 610041, China.

^2^Department of Rheumatology and Immunology, West China Hospital, Sichuan University, Chengdu, 610041, China.

^†^These authors contributed equally to this work.

*Correspondence: lunzhi.dai@scu.edu.cn (Dr. Lunzhi Dai); zhao.y1977@163.com (Dr. Yi Zhao)

**Supplemental table legends**

**supplemental Table S1**. The clinical information of RA and OA patients.

**supplemental Table S2**. Proteomes of RA and OA synovium. A total of 7227 proteins with FDR less than 1% in duplicates were quantified.

**supplemental Table S3**. List of 427 upregulated and 241 downregulated proteins in RA.

**supplemental Table S4**. List of 1260 intact *N*-glycopeptides derived from 481 *N*-glycosites on 334 *N*-glycoproteins with 2595 suggested glycan structures identified in RA and OA synovium.

**supplemental Table S5**. List of 67 upregulated and 9 downregulated intact *N*-glycopeptides in RA.

**supplemental Table S6**. List of 60 RA-specific and 4 OA-specific *N*-glycopeptides. RA-specific *N*-glycopeptides represented the peptides detected in no less than 75% of RA samples but not in any OA sample. In contrast, OA-specific *N*-glycopeptides represented the peptides detected in no less than 75% of OA samples but not in any RA sample. Among them, 29 RA-specific *N*-glycopeptides and 1 OA-specific *N*-glycopeptide were detected in all RA or OA samples but not in any sample in the other group.

**supplemental Table S7**. Antigenicity analysis results of 146 prototype peptides derived from *N*-glycopeptides with significant expression differences between RA and OA. Among them, 15 prototype peptides of 20 *N*-glycopeptides had an antigenic index greater than 1. The list of 146 prototype peptides derived from *N*-glycopeptides included **supplemental Tables S5** and **S6**, as well as *N*-glycopeptides detected in only one single sample of RA or OA but in no less than 75% of the samples in the other group.

**supplemental Table S8**. Representative gene lists for 9 identified immune cell clusters in RA.

**supplemental Table S9**. Spearman's rank correlation coefficient of the levels of 20 *N*-glycopeptides in **supplemental Table S7** with the enrichment scores of 9 immune cell types in **supplemental Table S8**. The levels of 5 *N*-glycopeptides were significantly correlated with the enrichment scores of certain immune cell types.
